# Additional evidence supports *GRM6* p.Thr178Met as a cause of congenital stationary night blindness in three horse breeds

**DOI:** 10.1111/vop.13151

**Published:** 2023-10-10

**Authors:** Elizabeth Esdaile, Kelly E. Knickelbein, Callum G. Donnelly, Michelle Ferneding, Monica J. Motta, Brett D. Story, Felipe Avila, Carrie J. Finno, Brian C. Gilger, Lynne Sandmeyer, Sara Thomasy, Rebecca R. Bellone

**Affiliations:** 1 Veterinary Genetics Laboratory, School of Veterinary Medicine, University of California, Davis, Davis, California, USA; 2 Department of Clinical Sciences, College of Veterinary Medicine, Cornell University, Ithaca, New York, USA; 3 Department of Population Health and Reproduction, School of Veterinary Medicine, University of California, Davis, Davis, California, USA; 4 Department of Surgical and Radiological Sciences, School of Veterinary Medicine, University of California, Davis, Davis, California, USA; 5 Department of Clinical Sciences, North Carolina State University, Raleigh, North Carolina, USA; 6 Department of Ophthalmology, School of Medicine, University of North Carolina, Chapel Hill, North Carolina, USA; 7 Department of Small Animal Clinical Sciences, Western College of Veterinary Medicine, University of Saskatchewan, Saskatoon, Saskatchewan, Canada; 8 Department of Ophthalmology & Vision Science, School of Medicine, University of California, Davis, Sacramento, California, USA

**Keywords:** allele frequency, CSNB, *CSNB2*, equine genetics, glutamate metabotropic receptor 6, night blind

## Abstract

Congenital stationary night blindness (CSNB) is an ocular disorder characterized by nyctalopia. An autosomal recessive missense mutation in glutamate metabotropic receptor 6 (*GRM6* c.533C>T, p.(Thr178Met)), called *CSNB2*, was previously identified in one Tennessee Walking Horse and predicted to reduce binding affinity of the neurotransmitter glutamate, impacting the retinal rod ON-bipolar cell signaling pathway. Thus, the first aim was to identify the allele frequency (AF) of *CSNB2* in breeds with reported cases of CSNB and breeds closely related to the Tennessee Walking Horse. The second aim was to perform ocular examinations in multiple breeds to confirm the link between genotype and CSNB phenotype. In evaluating 3518 horses from 14 breeds, the *CSNB2* allele was identified in nine previously unreported breeds. The estimated AF was highest in pacing Standardbreds (0.17) and lowest in American Quarter Horses (0.0010). Complete ophthalmic examinations and electroretinograms (ERG) were performed on 19 horses from three breeds, including one *CSNB2* homozygote from each breed. All three *CSNB2/CSNB2* horses had an electronegative ERG waveform under scotopic light conditions consistent with CSNB. The remaining 16 horses (seven *CSNB2/N* and nine *N/N*) had normal scotopic ERG results. All horses had normal photopic ERGs. This study provides additional evidence that *GRM6* c.533C>T homozygosity is likely causal to CSNB in Tennessee Walking Horses, Standardbreds, and Missouri Fox Trotting Horses. Genetic testing is recommended for breeds with the *CSNB2* allele to limit the production of affected horses. This study represents the largest across-breed identification of CSNB in the horse and suggests that this disorder is likely underdiagnosed.

## 1 ∣ INTRODUCTION

Congenital stationary night blindness (CSNB) is an inherited non-progressive, retinal disorder characterized by nyctalopia, the inability to see in dim light conditions, but with ostensibly unaffected vision in bright light. It is caused by faulty phototransduction of the ON-bipolar cells resulting in absent scotopic vision.^1-4^ Symptoms of CSNB in horses include the inability to find feed buckets at night, limited nocturnal movement, increased anxiety in the dark, and susceptibility to injury in low-light conditions. A full-field scotopic electroretinogram (ERG) is required to diagnose CSNB.^2^

Two genetic variants have been identified as causal to CSNB in horses. The first, termed *LP*, is a 1378 bp insertion of an endogenous retroviral long terminal repeat (LTR) in intron 1 of the transient receptor potential cation channel subfamily M member 1 (*TRPM1*) which impacts the ability of the ON-bipolar cells to depolarize due to a missing ion channel, inhibiting rod phototransduction.^1^ *LP* is an autosomal variant responsible for the incompletely dominant leopard complex spotting coat pattern and recessive CSNB found in Appaloosas and related breeds with *LP* spotting.^1,5,6^ Subsequent work identified variants in *TRPM1* to be causal for complete congenital stationary night blindness (cCSNB) in mice and humans.^2^

The second variant, termed *CSNB2* because it was the second cause of CSNB identified in horses, is also an autosomal recessive missense variant in metabotropic glutamate receptor 6 (*GRM6*) (NC_009157.3:g.2655618C>T, ENSECAT00000025528.4:c.533C>T, ENSE-CAP00000021237.3:p.Thr178Met).^3^ The *CSNB2* variant is predicted to alter the ligand-binding pocket of metabotropic glutamate receptor 6 (mGluR6) located on the dendritic tip of the ON-bipolar cell, impairing the binding ability of the neurotransmitter glutamate, inhibiting depolarization and thus halting rod phototransduction.^3^ Variants affecting *GRM6* are also recognized as causal for cCSNB in mice and humans, most commonly inhibiting trafficking and localization of mGluR6 to the dendric tip of the ON-bipolar cell.^2^ The *CSNB2* allele was initially reported in Tennessee Walking Horses, with an estimated allele frequency (AF) of 0.10 (*n* = 90); however, only one affected individual was identified and clinically evaluated in that study.^3^ Unlike *LP*, *CSNB2* has no reported effect on coat color.^3^

In addition to the aforementioned breeds, CSNB was previously clinically confirmed and reported in one Thoroughbred and one Paso Fino, with the Paso Fino possessing multiple congenital ocular disorders.^7^ Additionally, CSNB has been reported in Standardbreds and American Quarter Horses, but no clinical phenotyping or genotyping was described.^8,9^ Hack et al.^3^ did not detect the *CSNB2* allele in Friesians (*n* = 93), Thoroughbreds (*n* = 90), or American Quarter Horses (*n* = 90), therefore, the origin of *CSNB2* and AF in other breeds are unknown. The estimated AF of 0.10 in Tennessee Walking Horses suggests that *CSNB2* is likely responsible for night blindness in additional Tennessee Walking Horses, and the allele may be present in closely related breeds such as Standardbreds, Morgans, and American Saddlebreds.^10^ To date, little to no research on CSNB in any of these additional breeds has been performed.

Here, we aimed to further investigate *CSNB2* by determining the AF in several breeds, including those with previously reported cases of CSNB (Paso Finos, Thoroughbreds, American Quarter Horses, and Standardbreds)^3,7-9^ and those closely related to the Tennessee Walking Horse (Missouri Fox Trotting Horses, Morgans, Racking Horses, Rocky Mountain Horses, American Saddlebreds, Spotted Saddle Horses, and Standardbreds).^10-16^ Hackney Horses and Hackney Ponies were also investigated because they share a common ancestor (the Norfolk Trotter) with Standardbreds and Tennessee Walking Horses.^11^ Additionally, commercial testing at the UC Davis Veterinary Genetics Laboratory (VGL) identified *CSNB2* in two Miniature Horses; thus, we investigated the AF in Miniature Horses and Shetland Ponies due to the relatedness of these two breeds.^10^ Finally, we clinically evaluated 19 horses to investigate if *CSNB2* homozygosity is correlative with CSNB in the Tennessee Walking Horse, Standardbred, and Missouri Fox Trotting Horse.

## 2 ∣ MATERIALS AND METHODS

### 2.1 ∣ Animals and sample collection

Four thousand thirty-six samples were collected for DNA isolation or were used from banked DNA for this study. Hair samples from the mane or tail were collected from privately owned horses by the owner or a member of the research team with express written permission from the owner or agent (*n* = 83). Hair samples were also collected from university-owned animals (*n* = 9), were provided by the United States Trotting Association (USTA; *n* = 236), or were banked at the University of California—Davis, VGL (*n* = 1921). In addition, banked Thoroughbred samples used in this study were described previously in Elcombe et al.^17^ (*n* = 1787). Animals for clinical evaluation were selected based on *CSNB2* genotypes from the first aim and whose owners gave expressed written consent for inclusion in the study (*n* = 10) or were university-owned animals (*n* = 9).

### 2.2 ∣ Genotyping

DNA was isolated from hair follicles using the crude hair lysis protocol as previously described.^18^ Genotyping for *CSNB2* was conducted by the UC Davis VGL using the commercially available assay (https://vgl.ucdavis.edu/test/csnb-tennessee-walking-horse) or a custom-designed AgriSeq Targeted GBS panel using the IonChef and Ion GeneStudio S5 systems. *CSNB2* coordinates were retrieved from Hack et al.^3^ All horses positive for the *CSNB2* allele detected by the ArgiSeq Targeted GBS genotyping were also genotyped with the commercially available assay to validate types.

### 2.3 ∣ Allele frequency

Breed was assigned based on registration of individual or parents, and breed name was reported according to Vertebrate Breed Ontology.^19^ Standardbreds were grouped based on gait (trotter or pacer) as determined by race records or gait of sire for horses with no race records. This distinction was due to the previously reported population structure by gait phenotype.^20^ When parentage was known, individuals were filtered for at least two degrees of familial separation to calculate allele frequencies. Ninety-five percent confidence intervals for AF estimates were calculated using the modified Wald method.^21^

### 2.4 ∣ Clinical evaluation

Thirteen Standardbreds, three Missouri Fox Trotting Horses, and three Tennessee Walking Horses received complete ophthalmic examinations and photopic and scotopic ERGs. The neuro-ophthalmic examination included testing of the menace response, dazzle reflex, palpebral reflex, and pupillary light reflexes. Prior to further ophthalmic examination and dark adaption for the ERG, horses were sedated with 0.01–0.02 mg/kg of detomidine hydrochloride intravenously (Standardbreds and Missouri Fox Trotting Horses: Dormosedan^®^, Pfizer Animal Health USA; Tennessee Walking Horses: Dormosedan^®^, Orian Pharma, Pfizer Animal Health). Rebound tonometry was performed (TonoVet, Icare Finland Oy) followed by pharmacologic mydriasis with 0.2 mL of 1% tropicamide (Standardbreds and Missouri Fox Trotting Horses: Bausch and Lomb Inc.; Tennessee Walking Horses: Mydriacyl, Alcon, Canada). Auriculopalpebral nerve blocks were performed bilaterally with 1–2 mL of 2% lidocaine per site (Standardbreds and Missouri Fox Trotting Horses: Xylocaine^®^, APP Pharmaceuticals LLC; Tennessee Walking Horses: Bimeda-MTC Animal Health Inc.). Following mydriasis, the eyes were examined using slit-lamp biomicroscopy (SL-17 Biomicroscope, Kowa Company, Ltd), and indirect ophthalmoscopy (Standardbreds and Missouri Fox Trotting Horses: Keeler Vantage Plus; Tennessee Walking Horses: Heine Omega 200, Heine Instruments). Horses were then dark adapted for at least 20 min before the ERG.

The 13 Standardbreds and 3 Missouri Fox Trotters were examined at the William R. Pritchard Veterinary Medicine Teaching Hospital or the Center for Equine Health at the University of California, Davis, School of Veterinary Medicine, and received ERGs as follows. Needle electrodes (Stainless Steel Subdermal Needle Electrode, OcuScience) were placed subcutaneously with the reference electrode placed 3 cm posterior to the lateral canthus, the ground electrode placed anterior to the forelock, and the positive contact electrode (ERG-Jet, fabrinal) placed on the corneal surface. Proparacaine (1%, Sandoz) was applied topically to the ocular surface at least 1 min prior to placement of the positive electrode on the corneal surface with a coupling gel (Optixcare Eye Lube Plus, Aventix). Scotopic and photopic full-field ERG was performed with the RET*evet*^™^ device (LKC Technologies, Inc.) with firmware version 2.11.2 or 2.11.3. ERG testing consisted of using flash stimuli at 0.01, 3.0, and 10.0 cd·s/m^2^ in the dark-adapted state. After 10 min in the light-adapted state, a flash stimuli of 3.0 cd·s/m^2^ intensity was used, as well as a 30 Hz photopic flicker. Following the ERG, fluorescein staining of the cornea (BioGlo, HUB Pharmaceuticals LLC) was performed and a subset of horses received fundus photography (ClearView^™^ 2, Jorgensen Laboratories LLC).

The three Tennessee Walking Horses were examined at the Western College of Veterinary Medicine at the University of Saskatchewan and received ERGs as follows. Proparacaine (0.5%, Alcaine, Alcon Canada Inc.) was applied topically to the ocular surface at least 1 min prior to placement of a corneal DTL^™^ microfiber electrode (DTL Plus Electrode, Diagnosys LLC) which was attached at the medial and lateral canthi with glue (Krazy Glue, Elmer Products Canada Corp.). Platinum subdermal needle electrodes (Cadwell Low Profile Needle Electrodes, Cadwell Laboratories) were placed subdermally 3 cm posterior to the lateral canthus as reference and subdermally over the occipital region as ground. The ERGs were elicited with a white xenon strobe light and recorded with a Sierra Summit^™^ (Cadwell Industries, Inc.). Scotopic flash ERGs (3.0 cd·s/m^2^, background 0.0 cd/m^2^) were completed in each eye.

Measurements for all horses were recorded and evaluated utilizing the respective ERG machine manufacturer's software. The ImageJ 2.9.0 Figure Calibration plugin was used to identify the ERG tracing coordinates for the *CSNB2/CSNB2* Tennessee Walking Horse from the manufacturer's report.^22^ These coordinates and raw data from the RET*evet* device were visualized with GraphPad Prism Version 9.5.0 for publication (GraphPad Software, Inc). CSNB diagnoses were determined by Diplomates of the American College of Veterinary Ophthalmologists based on complete ophthalmic examinations and ERG results.

## 3 ∣ RESULTS

### 3.1 ∣ Allele frequency

Five hundred and fifteen horses were excluded from the AF calculations due to relatedness or lacking breed registration. The estimated AF of *CSNB2* was determined in 14 breeds from a total of 3518 horses (Table 1). The *CSNB2* allele was present in nine breeds, ranging in frequency from 0.0010 in American Quarter Horses (*n* = 486) to 0.17 in pacing Standardbreds (*n* = 110; Table 1). The *CSNB2* allele was not detected in trotting Standardbreds (*n* = 70), Thoroughbreds (*n* = 1787), Hackney Horses (*n* = 47), Hackney Ponies (*n* = 44), and Shetland Ponies (*n* = 99; Table 1).

### 3.2 ∣ Ophthalmic examinations and electroretinograms

Three Tennessee Walking Horses (1 *N/N*, 1 *CSNB2/N*, 1 *CSNB2/CSNB2*), 10 pacing Standardbreds (5 *N/N*, 4 *CSNB2/*N, *1 CSNB2/CSNB2*), 3 trotting Standardbreds (3 *N/N*), and 3 Missouri Fox Trotting Horses (2 *CSNB2/N*, 1 *CSNB2/CSNB2*) received complete ophthalmic examinations and ERG (Table 2). Incidental abnormalities detected during the ophthalmic examination can be found in Table S1. Given that those anomalies found in more than one horse were found in both night-blind and non-night-blind horses, these anomalies are unlikely to be related to the *GRM6* mutation.

**FIGURE 1:**
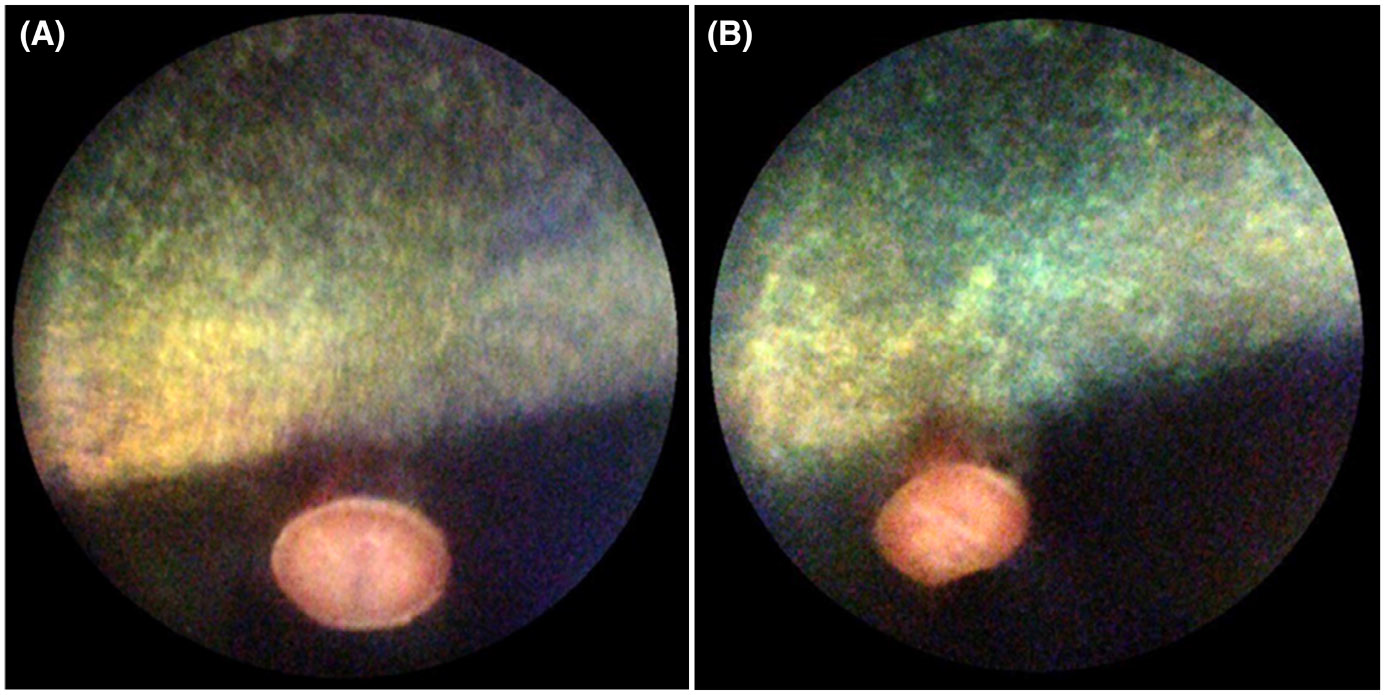
Representative fundus images. (A) 3-year-old *CSNB2/N* Standardbred unaffected by CSNB and (B) 5-year-old *CSNB2/CSNB2* Standardbred mare affected by CSNB. Both show normal fundic appearance with no observable abnormalities.

**FIGURE 2:**
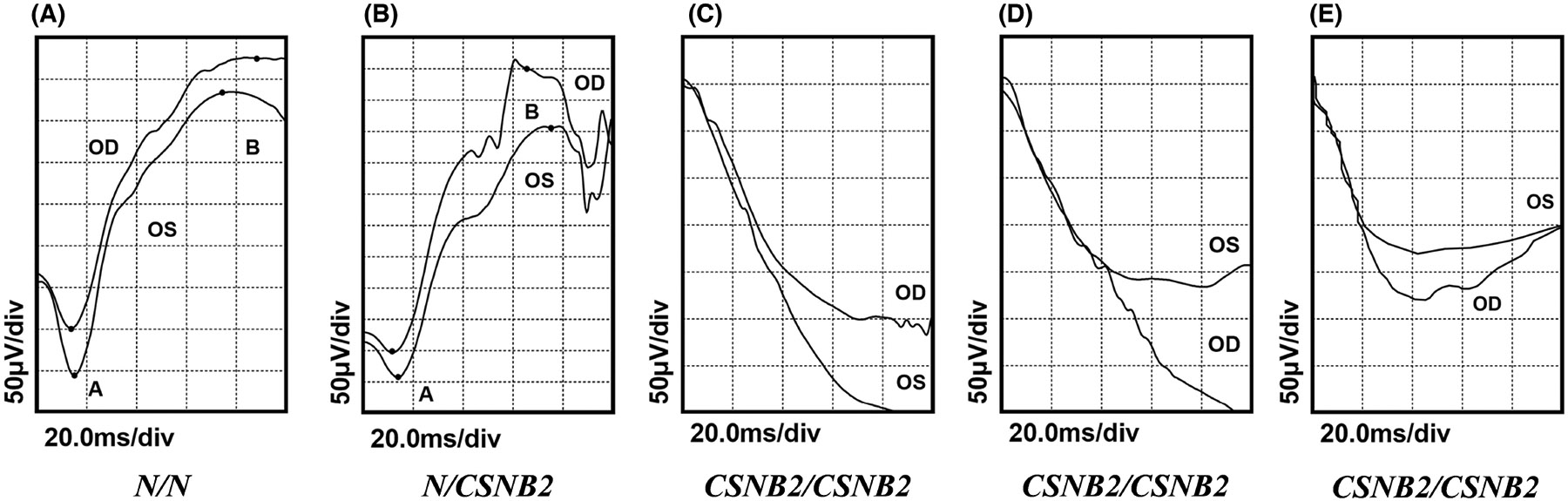
Representative scotopic ERG tracings. Normal scotopic waveforms were bilaterally detected in (A) a 29-year-old Standardbred *CSNB2: N/N* horse and (B) a 16-year-old *N/CSNB2* horse. “A” and “B” labels mark the A wave and B wave, respectively. Electronegative ERG waveforms were detected in all three *CSNB2/CSNB2* horses (C–E). (C) 5-year-old Standardbred, (D) 20-year-old Missouri Fox Trotting Horse, and (E) 12-year-old Tennessee Walking Horse. In all panels, results for the right and left eyes are denoted as “OD” and “OS,” respectively. (A–D) were recorded on a RET*evet* device (LKC Technologies, Inc.) under the following parameters: flash: 3.0 cd·s/m^2^, chromaticity (0.33, 0.33) at 0.1 Hz, background: 0.0 cd/m^2^. (E) was recorded on a Sierra Summit^™^ (Cadwell Industries, Inc.) under the following parameters 3.0 cd·s/m^2^, background 0.0 cd/m^2^. Each tracing starts at (0, 0).

Multiple horses had age-related and incidental anterior segment findings, as detailed in Table S1. Apart from peripapillary bullet hole lesions (2 *N/N*, 1 *CSNB1/N*), indirect ophthalmoscopy findings were unremarkable in all horses (Figure 1). Regardless of *CSNB2* genotype, all horses had normal photopic ERG results and all *N/N* (*n* = 9) and *CSN-B2/N* (*n* = 7) horses had normal scotopic ERG results indicating they were unaffected by CSNB (Table 2, Figure 2). All three *CSNB2/CSNB2* individuals were clinically affected by CSNB as identified by electronegative scotopic ERGs (Table 2, Figure 2).

## 4 ∣ DISCUSSION

These findings build upon previous research describing the identification of missense variant in *GRM6* (p.(Thr178Met))^3^ and suggest that homozygosity (denoted as *CSNB2/CSNB2*) is likely causal for CSNB in three horse breeds. Further functional investigations are required to confirm the molecular causality of *GRM6* p.(Thr178Met).

Additionally, we identified the *CSNB2* variant in two breeds previously reported in the literature to have cases of CSNB: Standardbreds at a relatively high AF in the pacing subpopulation and American Quarter Horses at a very low AF. We also detected the *CSNB2* allele in Miniature Horses, a breed that segregates for leopard complex spotting pattern^5^ and thus also likely has cases of CSNB caused by a mutation in *TRPM1* and *CSNB2*. Therefore, given that *LP* and *CSNB2* are present in Miniature horses, testing for these variants is important for breeding and management. It is yet unknown how vision is impacted in horses with both *LP* and *CSNB2*, which should be evaluated in this breed. *CSNB2* was also detected at varying frequencies in breeds with no published reports of CSNB (Missouri Fox Trotting Horses, Morgans, Racking Horses, Rocky Mountain Horses, American Saddlebreds, and Spotted Saddle Horses). The presence of *CSNB2* and moderate allele frequencies in multiple breeds but the lack of clinical evidence of affected horses from these breeds in the literature suggests that CSNB is underdiagnosed across horse breeds. Additional clinical screening of horses from a wide variety of breeds may help to shed further light on the prevalence of CSNB in horses.

Many of the breeds with *CSNB2* have intertwined histories. The Morgan, the Norfolk Trotter, Canadian Pacer, and Narragansett Pacer are all reported to have influenced the foundation of the Tennessee Walking Horse, American Saddlebred, and Standardbred.^11^ Additionally, Morgans, Standardbreds, American Saddlebreds, and Tennessee Walking Horses heavily influenced the development of the Missouri Fox Trotting Horse, Rocky Mountain Horse, Spotted Saddle Horse, and Racking Horse.^12-16^ These reported relationships make it unsurprising that *CSNB2* was identified in these breeds.

Although the American Quarter Horse was predominantly influenced by Thoroughbreds and descendants of Spanish horses,^10^ Morgan ancestry is reportedly present in the breed.^11^ This Morgan ancestry may be responsible for the low *CSNB2* AF identified in the American Quarter Horse in this study and the lack of detecting the allele in Quarter Horses in the previous study.^3^

Finding the *CSNB2* allele in Miniature Horses was unexpected, as there is no closely reported relationship between the Miniature Horse and breeds with the *CSNB2* allele. Some reports suggest that horses from the British Isles influenced the foundation of Miniature Horses, Morgans, the Narragansett Pacer, and their descendants. Pit ponies (a term that includes both horses and ponies) from Britain and mainland Europe were also used in Appalachian mines and may have influenced the American Miniature Horses. Additionally, gene flow may be present in the Miniature Horse breed, as the largest registry of Miniature Horses, the American Miniature Horse Association was not founded until 1978, and the studbook remains open to individuals within the breed standard.^23^

We did not identify the *CSNB2* allele in Thoroughbreds or Paso Finos, two breeds with clinically confirmed cases of CSNB.^7^ Given the extensive number of variants causal to CSNB in humans, it is possible that additional variants are responsible for CSNB in these breeds. This is supported by the different reported scotopic ERG waveform of the Thoroughbred compared with the four *CSNB2/CSNB2* horses and the alternating head tilt, poor vision in bright light, and bilateral dorsomedial strabismus of the Paso Fino colt that was not present in *CSNB2/CSNB2* horses.^2,3,7^ Genotyping affected individuals in these breeds is required to test these hypotheses further.

The high AF of *CSNB2* (0.17) in pacing Standardbreds but absence in trotting Standard-breds was also unexpected. While recent studies suggest that these two populations show moderate genetic differentiation,^20^ this finding suggests that the *CSNB2* allele may be in linkage disequilibrium (LD) with a variant under positive selection in pacers or is being negatively selected in the trotting population. For example in pacing Standardbreds, the group of horses with the highest detected *CSNB2* AF, a gain of function mutation in nearby genes *CANX* and/or *CPLX* may be in LD with *CSNB2* and may positively impact pacing gait performance as knockout mice for these genes show gait abnormalities.^24-26^ Thus, more work is necessary to investigate whether variants near *GRM6* impact performance and other phenotypes and whether these contribute to the varied *CSNB2* AF between populations.

Additionally, given that many Standardbred races are held at night, more work is needed to determine if this allele and disorder impact racing performance in this breed. Furthermore, the estimated AF of 17% suggests that 3% of pacing Standardbreds, or 440 of the 14 679 pacing standardbreds who raced in North America in 2022, are homozygous for *CSNB2*.^27^ Careful consideration of training and racing is advised for those pacing Standardbreds that are homozygous and thus night blind.

## 5 ∣ CONCLUSION

These data support that *CSNB2* homozygous horses are night blind, increasing the number of examined and confirmed CSNB affected *CSNB2/CSNB2* horses in the literature from one to four (two Tennessee Walking Horses, one Standardbred, and one Missouri Fox Trotting Horse). Because the affected horses are different breeds, we suspect that all horses who are homozygous for this allele will be night blind. Thus, we recommend *CSNB2* genetic testing in breeds where the *CSNB2* allele was identified in this study and clinical evaluation of those that test homozygous (*CSNB2/CSNB2*). Additionally, genotyping horses from breeds with the *CSNB2* allele can be used to make informed breeding decisions to avoid producing affected offspring. Finally, while horses identified clinically as night blind should be evaluated for both *LP* and *CSNB2*, we recognize that unidentified variants in the ON-bipolar cell phototransduction cascade may cause similar phenotypes, and once the known alleles are ruled out, these cases should be evaluated by similar approaches used previously by Hack et al.^3^

## Supplementary Material

Supplementary table

Additional supporting information can be found online in the Supporting Information section at the end of this article.

## Figures and Tables

**TABLE 1:** *CSNB2* genotypes, allele frequencies, and 95% confidence intervals (CI) by breed.

Breed	Vertebrate breed ontology ID^19^	*n*	*N/N*	*CSNB2/N*	*CSNB2/CSNB2*	*CSNB2* allele frequency (95% CI)^a^
Miniature Horse	VBO:0000896	99	97	2	0	0.010 (<0.0010–0.039)
Missouri Fox Trotting Horse	VBO:0011833	89	76	11	2	0.084 (0.051–0.14)
Morgan	VBO:0001022	330	321	9	0	0.014 (0.0068–0.026)
American Quarter Horse	VBO:0001057	486	485	1	0	0.0010 (<0.00010–0.0064)
Racking Horse	VBO:0016883	58	46	12	0	0.10 (0.059–0.17)
Rocky Mountain Horse	VBO:0011836	74	73	1	0	0.0068 (<0.00010–0.041)
American Saddlebred	VBO:0000898	92	91	1	0	0.0054 (<0.0001–0.022)
Spotted Saddle Horse	VBO:0016884	55	44	11	0	0.10 (0.055–0.17)
Standardbred (pacer)	VBO:0000899	110	75	32	3	0.17 (0.13–0.23)
Hackney Horse	VBO:0000976	47	47	0	0	0.00
Hackney Pony	VBO:0000977	44	44	0	0	0.00
Paso Fino	VBO:0001040	78	78	0	0	0.00
Shetland Pony	VBO:0001066	99	99	0	0	0.00
Standardbred (trotter)	VBO:0000899	70	70	0	0	0.00
Thoroughbred	VBO:0001083	1787	1787	0	0	0.00

a CI are only reported for those breeds in which the *CSNB2* allele was detected.

**TABLE 2:** *CSNB2* genotype and congenital stationary night blindness (CSNB) disease status.

Breed	CSNB unaffected, *CSNB2: N/N*	CSNB unaffected, *CSNB2: CSNB2/N*	CSNB affected, *CSNB2: CSNB2/CSNB2*
Tennessee Walking Horse	1	1	1
Standardbred (Pacer)	5	4	1
Standardbred (Trotter)	3	0	0
Missouri Fox Trotting Horse	0	2	1
Total	9	7	3
